# Conceptual knowledge shapes visual working memory for complex visual information

**DOI:** 10.1038/s41598-022-12137-0

**Published:** 2022-05-16

**Authors:** Chris R. Sims, Rachel A. Lerch, John A. Tarduno, Robert A. Jacobs

**Affiliations:** 1 Department of Cognitive Science, Rensselaer Polytechnic Institute, Troy, NY 12180 USA; 2 Department of Psychology, University of Texas at Austin, Austin, TX 78712 USA; 3 Department of Earth and Environmental Sciences, University of Rochester, Rochester, NY 14627 USA; 4 Department of Brain and Cognitive Sciences, University of Rochester, Rochester, NY 14627 USA

**Keywords:** Human behaviour, Working memory

## Abstract

Human visual working memory (VWM) is a memory store people use to maintain the visual features of objects and scenes. Although it is obvious that bottom-up information influences VWM, the extent to which top-down conceptual information influences VWM is largely unknown. We report an experiment in which groups of participants were trained in one of two different categories of geologic faults (left/right lateral, or normal/reverse faults), or received no category training. Following training, participants performed a visual change detection task in which category knowledge was irrelevant to the task. Participants were more likely to detect a change in geologic scenes when the changes crossed a trained categorical distinction (e.g., the left/right lateral fault boundary), compared to within-category changes. In addition, participants trained to distinguish left/right lateral faults were more likely to detect changes when the scenes were mirror images along the left/right dimension. Similarly, participants trained to distinguish normal/reverse faults were more likely to detect changes when scenes were mirror images along the normal/reverse dimension. Our results provide direct empirical evidence that conceptual knowledge influences VWM performance for complex visual information. An implication of our results is that cognitive scientists may need to reconceptualize VWM so that it is closer to “conceptual short-term memory”.

## Introduction

In recent decades, cognitive scientists have conducted scores of experiments probing the properties of human visual working memory (VWM), the memory store people use to maintain the visual features of objects and scenes. It is commonly thought that VWM is utilized for planning eye movements and facilitating motor control, or when searching for an object in a cluttered scene^1,2^. Experiments have attempted to shed light on the properties of VWM representations, the limits of what VWM can represent, the regularities of VWM errors, and the task-dependent nature of VWM performance, among many other issues^3^.

From this wealth of research, it is clear that VWM is influenced by bottom-up information (i.e., information derived from the pattern of light that enters an observer’s eyes), such as information regarding objects’ locations, shapes, sizes, orientations, and colors. While VWM has traditionally been studied as a distinct system from episodic or semantic memory, there is accumulating evidence to suggest that VWM is also influenced by top-down information, such as conceptual knowledge. However, the majority of existing studies on the role of top-down influence on VWM suffer from one or more limitations that limit the strength of their conclusions. The present paper reports an experiment and a novel methodology to address these limitations, and provides direct evidence for the influence of conceptual knowledge on human VWM.

One limitation common to existing studies is the use of very simple stimuli, such as oriented bars or colored squares. Even with such ‘impoverished’ stimuli, there is moderate evidence that category knowledge exerts influence over VWM performance. For example, culturally-defined color categories bias the perception and memory of color^4–8^, and VWM for line orientation is particularly sensitive around vertical and horizontal angles, known as the oblique effect^9–11^. Other studies have shown that VWM is sensitive to the statistical structure of stimuli, by exhibiting a central-tendency bias^12,13^, or adaptation to the variance of the stimulus distribution^14,15^. Using EEG and fMRI decoding techniques, it has been argued that categorical biases are present in early visual cortical representations^16,17^, though Luu and Stocker^18^ argued that top-down categorical information does not influence VWM directly, but rather influences subsequent sensory recall and inference processes.

Despite the accumulating evidence for top-down effects provided by these studies, an important limitation remains. Humans possess rich conceptual knowledge about the world, and the use of meaningless or overly simplified visual stimuli greatly limits the potential contribution of such knowledge^19^. Hence it is possible that top-down information exhibits a much greater degree of influence than currently understood.

Alternately, a smaller number of studies have utilized rich, complex stimuli. However, in such cases it is more difficult to measure precisely how VWM performance is altered by the presence of top-down information, due to several factors. First, complex stimuli exhibit variation along many dimensions, and thus probes of memory performance may be sparsely sampled in a high-dimensional stimulus space. Second, complex stimuli can present an additional challenge, in that peoples’ conceptual knowledge about complex visual information can be difficult to manipulate in an experimental setting. For example, several studies of category effects in VWM have used human faces as stimuli, a domain where humans naturally possess deep perceptual expertise and rich conceptual knowledge^20–22^. However, it is difficult or impossible to compare performance between groups that differ in their conceptual knowledge of human faces in general.

A limited number of studies have also shown evidence for the influence of category knowledge on VWM using other complex stimuli. Brady et al.^23^ found that long encoding times aided VWM performance for real-world objects but not for simple stimuli. Asp et al.^24^ reported that VWM performance was improved when visually ambiguous stimuli were perceived as meaningful. Conci et al.^25^ found better VWM performance when color-shape combinations formed meaningful stimuli (e.g., real flags of European countries) than when they formed meaningless stimuli (“fake” flags). Hu and Jacobs^26^ found greater VWM performance using real-world objects when all objects in a display belonged to the same conceptual category. Using natural image stimuli, Liu et al.^27^ argued for visual and semantic representations in VWM based on neural (intracranial electroencephalography; iEEG) data. However, none of these studies experimentally manipulated peoples’ conceptual knowledge. In summary, while existing studies are consistent with the influence of top-down knowledge on VWM, without a direct experimental manipulation they cannot assign a causal role to the influence of such information.

In this article, we report an experiment focusing on the role of categorical knowledge in human VWM. Our experiment uses complex meaningful stimuli, but importantly, we utilize stimuli for which our participants have limited pre-experimental conceptual knowledge (animations of geologic faults). This enables us to train different groups of subjects with different types of conceptual knowledge. This combination of features in our experiment enables a particularly clear demonstration of the influence of conceptual knowledge on visual working memory. Using a change-detection paradigm, perhaps the most common paradigm for studying VWM in the scientific literature, the experiment asks whether VWM performance is altered when a person views a scene that they know how to categorize (e.g., a geologic scene that is an exemplar of a particular category). In brief, we find that the answer is “yes”: observers are more sensitive to visual changes when they cross known category boundaries.

In addition to this empirical contribution, this paper also makes a methodological contribution. The more realistic scenario studied here (relative to earlier VWM studies) leads to an interesting data analysis challenge. In our experiment, it was not possible to densely sample the entire space of possible stimuli because this space is too big. Although our experiment sparsely sampled this space, it is desirable to make predictions about people’s performance for all possible stimuli. To this end, we analyzed the experimental data using a Bayesian model with a Gaussian Markov random field (GMRF) prior distribution^28^. This statistical model assumes that people exhibit similar performances with similar stimuli. In other words, it assumes dependencies in people’s performances at neighboring regions of the stimulus space. We believe that our Bayesian model provides a useful illustration of how sparsely-sampled datasets can be successfully analyzed, a scenario that will become more prevalent as the study of real-world situations becomes more commonplace in the field of cognitive science.

## Results

Many existing studies of VWM use simple, artificial stimuli, such as oriented bars or colored squares. An advantage of the use of these stimuli is that they allow investigators to conduct carefully controlled experiments that yield datasets which are easy to analyze. A disadvantage is that performance with these simple stimuli may not be representative of performance with complex, real-world stimuli. The experiment reported here attempted to achieve a compromise by making use of simplified versions of real-world stimuli. In particular, it used animations of realistic geologic scenes of the type that are commonly studied in college geoscience courses.

**Figure 1:**
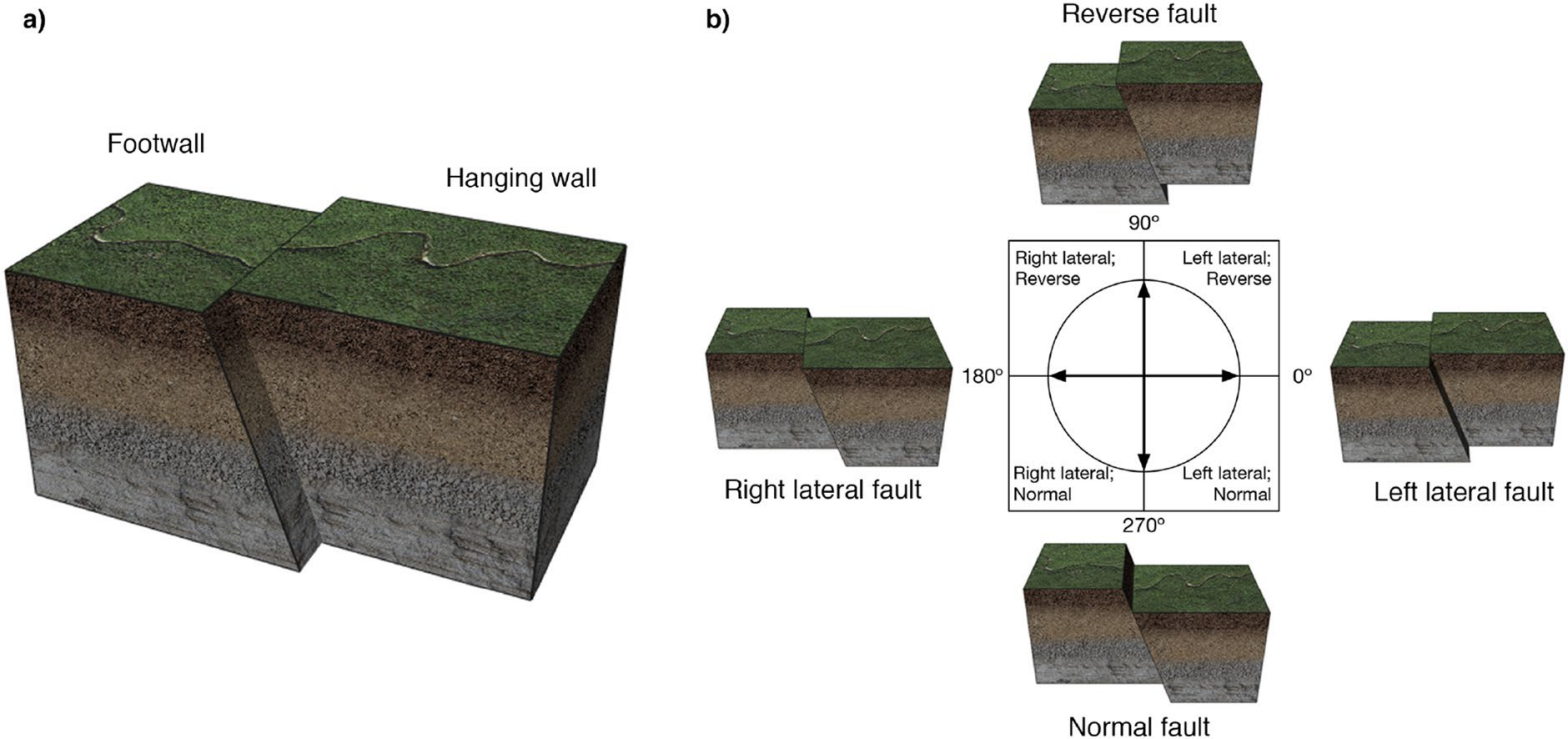
(**a**) An example of a geologic fault used as a stimulus in the experiment. It illustrates a fault plane separating two sections of Earth’s crust, called fault blocks. One of the blocks is labeled as the ‘footwall’ and the other is labeled as the ‘hanging wall’. There is a displacement in rock types on each side of the fault, indicating that the blocks have moved relative to each other. The direction of displacement between blocks is referred to as the rake angle. (**b**) Faults can be categorized according to their rake angle, with four ‘prototypical’ fault categories illustrated. A left lateral fault ($0^\circ$ rake angle) has the hanging wall shifted laterally (to the left) relative to the footwall. A reverse fault ($90^\circ$ rake angle) has the hanging wall elevated relative to the footwall. A right lateral fault ($180^\circ$ rake angle) has the hanging wall shifted right relative to the footwall. A normal fault ($270^\circ$ rake angle) has the hanging wall sunken relative to the footwall. Note that fault categories are partially overlapping; a fault with $30^\circ$ rake angle would be categorized as both a left lateral and a reverse fault. (Figure generated using Adobe Illustrator 2021 and Adobe Photoshop 2021).

Scenes depicted geologic *faults*, or fractures or discontinuities in a volume of rock along which there has been displacement (see Fig. 1). A fault might be categorized as a *normal* or *reverse* fault if there has been downward or upward relative movement of the blocks on either side of the fault. In addition, a fault might be categorized as a *left lateral* or *right lateral* fault if there has been left or right relative movement of the blocks. The relative movement of the blocks can be quantified by the *rake angle*. Importantly, a fault can belong to two categories. For instance, a fault with a rake angle of 30$^\circ$ is categorized as both a reverse fault and a left lateral fault because movement along this fault has both upward and leftward components (see “Methods” for further details).

We recruited three groups of participants for our experiment. Each group watched a series of instructional videos teaching them about the basic properties of geologic faults. The groups differed only in the content of this instruction. The first group was instructed on the basic properties of geologic faults, and was then taught about the distinction between left lateral and right lateral faults. The second group was instead taught about the distinction between normal faults and reverse faults. The third group (henceforth referred to as the control condition) was instructed about some basic properties of faults but was not given any information on different categories of faults. Following training, participants in all three groups performed a change detection experiment. On each trial, a participant viewed an animation of one fault, referred to as the “memory” stimulus, followed by an animation of a second fault, referred to as the “probe” stimulus. The participant then judged whether the faults depicted in the memory and probe were the same or different.

**Figure 2:**
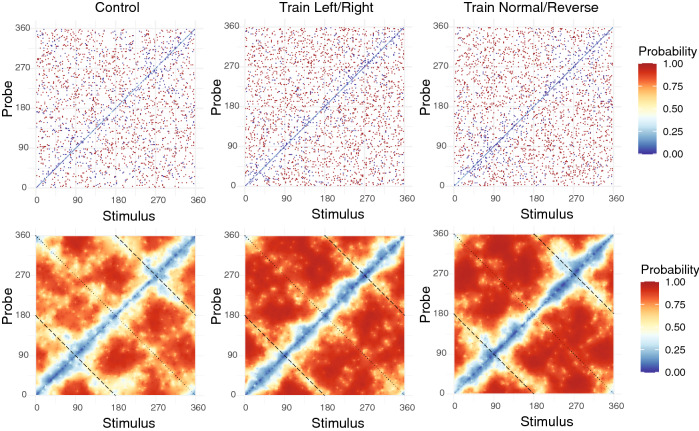
Visual change detection performance. Top row: Raw data from each of the three experimental conditions. The horizontal and vertical axes of a graph indicate the rake angles of the memory and probe stimuli, respectively. The color of a plot marker corresponds to the probability of reporting a change trial. Bottom row: Posterior mean change detection probability estimated via Bayesian model. The dashed and dotted lines overlaid on the graph correspond to “mirror stimuli” (see main text). (Figure generated using R version 4.0.3 and Adobe Illustrator 2021).

The primary question addressed by this experiment is whether and how categorical knowledge of geologic faults impacts performance on the change detection task. Figure 2, top row, shows the complete dataset from all three conditions of the experiment. The horizontal and vertical axes of the graph indicate the rake angles of the memory and probe stimuli, respectively. Plot markers are colored according to the probability that a memory–probe pair was judged as a ‘change’ trial. By design of the experiment, half of all trials were change trials, and the remaining half were no-change trials. Results from no-change trials are visible as the values along the main diagonal in each graph. As expected, these trials were relatively unlikely to be judged as change trials (indicated by the dark blue color). The off-diagonal plot markers correspond to change trials.

The large number of possible memory–probe pairs (32,400 combinations; see “Methods”) led to an interesting data analysis challenge. In our experiment, it was not possible to densely sample the entire space of possible stimuli because this space is too big. Although our experimental stimuli sparsely sampled this space, it is desirable to make predictions about people’s performance for all possible stimuli. To this end, we analyzed the experimental data using a Bayesian model with a Gaussian Markov random field (GMRF) prior distribution^28^ that assumes that people exhibit similar performances with similar stimuli. In other words, it assumes dependencies in people’s performances at neighboring regions of the stimulus space (see “Methods”).

Figure 2, bottom row, shows the results obtained using the Bayesian model. These figures show the posterior mean probability of reporting a change trial for the two training conditions. Importantly, the GMRF prior allows for ‘sharing statistical strength’^29^ between observations, and in particular assumes that trials with similar memory and probe rake angles will exhibit similar change detection performance. As can be seen in the graphs, this also enables interpolating change detection performance even to stimulus combinations that were not directly observed in the dataset.

The Bayesian analysis reveals that no-change trials had a low probability of being reported as change trials. Critically, the analysis also reveals interesting structure in the dataset for change trials. In particular, human observers have greater difficulty detecting a change for certain stimulus combinations compared to others, and change detection performance also appears to differ depending on the observer’s category knowledge.

**Figure 3:**
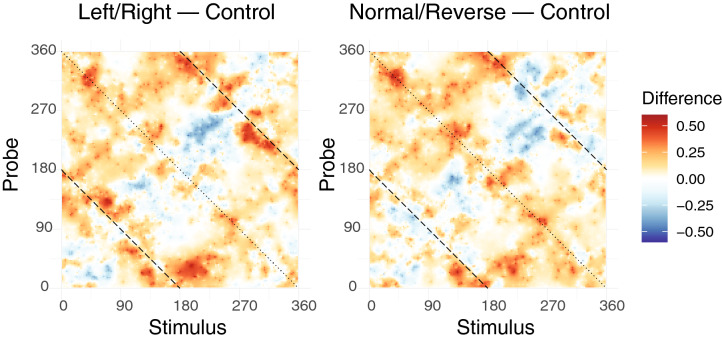
Difference in visual change detection probability, relative to the control condition. The left figure shows the probability of reporting a change in the condition trained in left/right faults, minus the corresponding probability for the control condition. Hence positive values (warm colors) indicate a stimulus-specific increase in change detection probability, while negative values (cool colors) indicate a decrease in change detection probability. The right figure shows the normal/reverse training condition relative to the control condition. The dashed and dotted lines correspond to “mirror stimuli” (see main text). (Figure generated using R version 4.0.3 and Adobe Illustrator 2021).

To render these differences more salient, Fig. 3 shows the posterior mean difference in change detection probability. In short, this figure provides a detailed map of precisely how differences in conceptual knowledge translated to differences in VWM performance. In each, warm colors indicate stimuli where changes are more likely to be detected, and cool colors indicate stimuli where changes were less likely to be detected. The left figure compares performance in the “Train Left/Right” condition relative to the control condition. The right figure compares the “Train Normal/Reverse” condition to the control condition. The figure illustrates that changes in VWM performance in our experiment cannot be described by an overall improvement or decline. Instead, some types of visual changes were rendered more salient to observers while others were less likely to be detected, depending on their conceptual knowledge. Superimposed on Fig. 2 (bottom row) and Fig. 3 are dotted and dashed lines orthogonal to the main diagonal. Based on visual inspection of the figures, it is apparent that these memory–probe combinations in particular were often difficult for participants to judge correctly and, importantly, were also sources of performance differences between training conditions.

**Figure 4:**
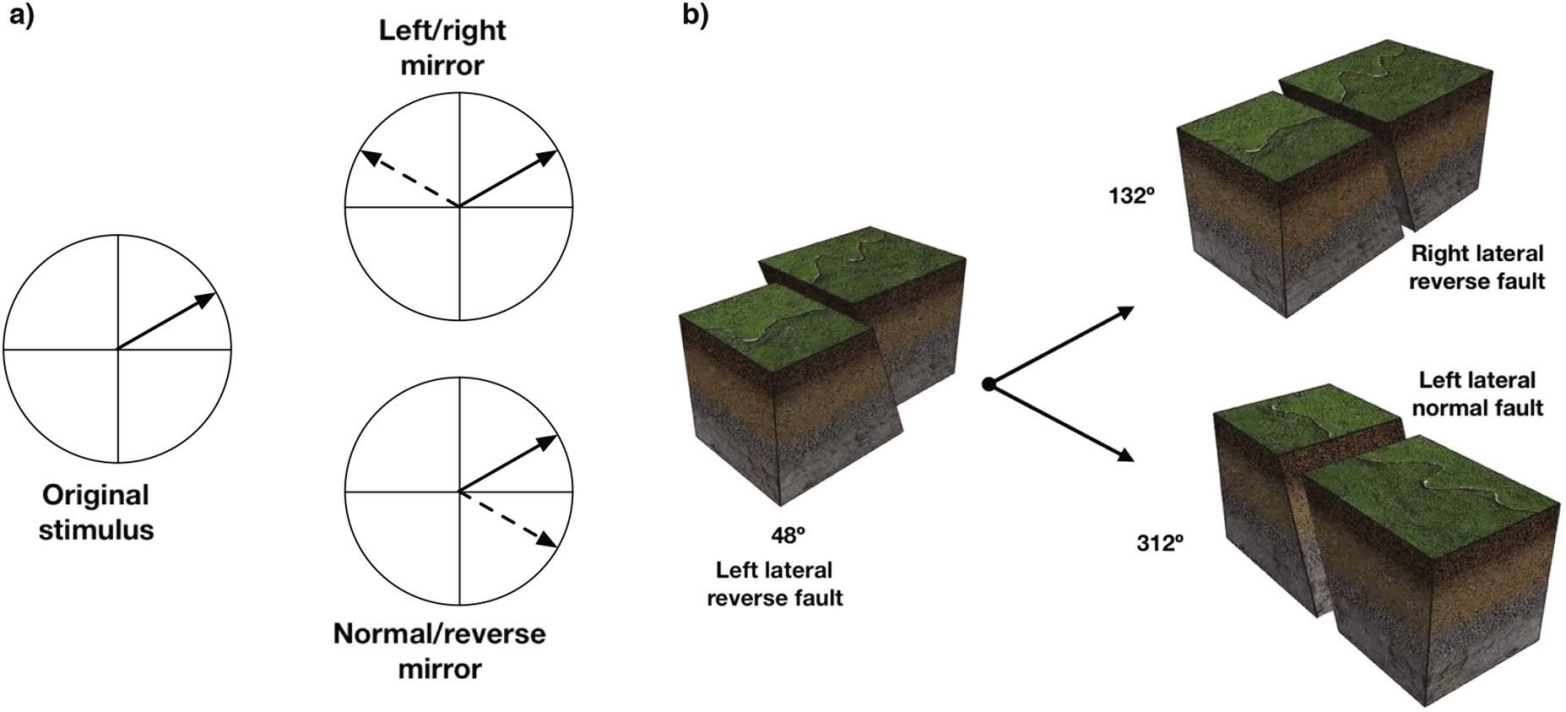
(**a**) Every fault defined by a particular rake angle has two corresponding “mirror images” that represent their reflection around the normal/reverse and left/right axes. (**b**) Illustration of mirror stimuli for a fault with a rake angle of 48$^\circ$. (Figure generated using Adobe Illustrator 2021 and Adobe Photoshop 2021).

Interestingly, the origin of this phenomenon lies in the visual symmetry of the geologic fault stimuli. Every geologic fault defined by a particular rake angle has two “mirror image” faults, defined by reflecting the rake angle around either the left/right fault axis or the normal/reverse axis. This effect is easily visualized, as in Fig. 4. The dashed lines overlaid on Figs. 2 and 3 correspond to trials where the memory and probe stimuli were left/right mirror images, and the dotted line corresponds to trials with normal/reverse mirror symmetry. Thus, the results in Fig. 3 illustrate that participants trained in the categorical distinction between left and right lateral faults were more likely to detect a change when the memory and probe were mirror images along the left/right lateral dimension. Similarly, participants trained to categorize normal/reverse faults were more likely to detect a change when the memory and probe were mirror images along the normal/reverse dimension. These results provide direct evidence for top-down effects on VWM performance, but also illustrate that complex visual information and rich conceptual knowledge can interact in complex ways that may be obscured when using simple visual stimuli. Importantly, these complex interactions were only apparent by virtue of our novel Bayesian analysis.

The results so far have focused on the effects of training at the level of individual memory–probe configurations with mirror symmetry. We now turn to the analysis of broader hypotheses. In particular, we hypothesized that by training participants in a categorial distinction, visual changes that crossed a category boundary would be rendered more salient compared to participants who were presented with the same exact visual stimulus but were naive to the relevant categorical distinction.

Memory–probe combinations can be classified depending on whether or not they cross the two categorical distinctions (i.e., normal/reverse, left/right) trained in our experiment. We define a “critical” trial to be a change trial on which the memory stimulus belonged to one category but the probe stimulus belonged to another. We hypothesized that critical trials would be more detectable, but only for participants trained in the relevant categorical distinction.

**Figure 5:**
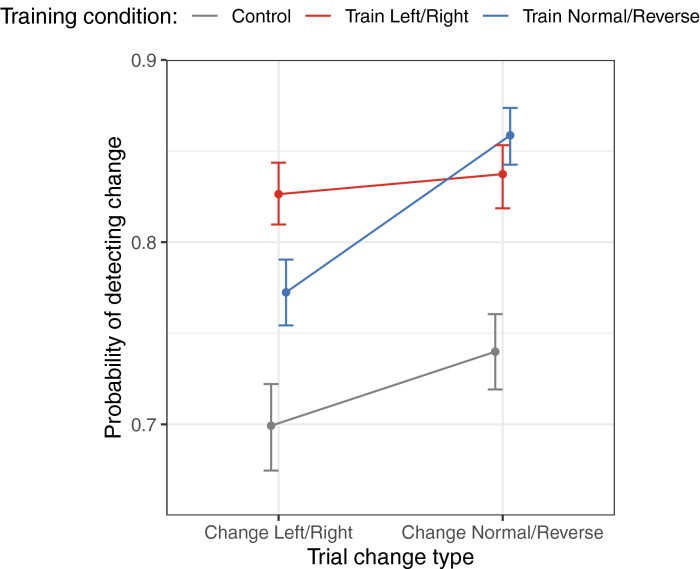
Performance on change trials that cross category boundaries, as a function of boundary type and experimental training condition. We predicted that change detection performance would be highest when the visual change corresponded to a known categorical distinction. Error bars correspond to 95% highest density credible intervals (HDCI) around the mean. Values in parentheses indicate the 95% HDCI for the difference between conditions. (Figure generated using R version 4.0.3).

To evaluate this hypothesis, we computed the posterior mean change detection probability across all critical trials, and examined how change detection performance differed between the three training conditions. These results are shown in Fig. 5. Several interesting results can be observed. First, for participants in the control condition (who received no category training) changes that crossed the normal/reverse category boundary were more detectable than changes that crossed the left/right category boundary. For participants who received training in the distinction between normal and reverse faults, visual changes that crossed this boundary were especially salient. Participants who received training in the left/right fault distinction exhibited superior ability to detect changes across this categorical distinction. These results are noteworthy because fault category knowledge was not directly relevant for the change detection task. Lastly, both groups that received training in fault categories exceeded change detection performance in the control condition.

**Figure 6:**
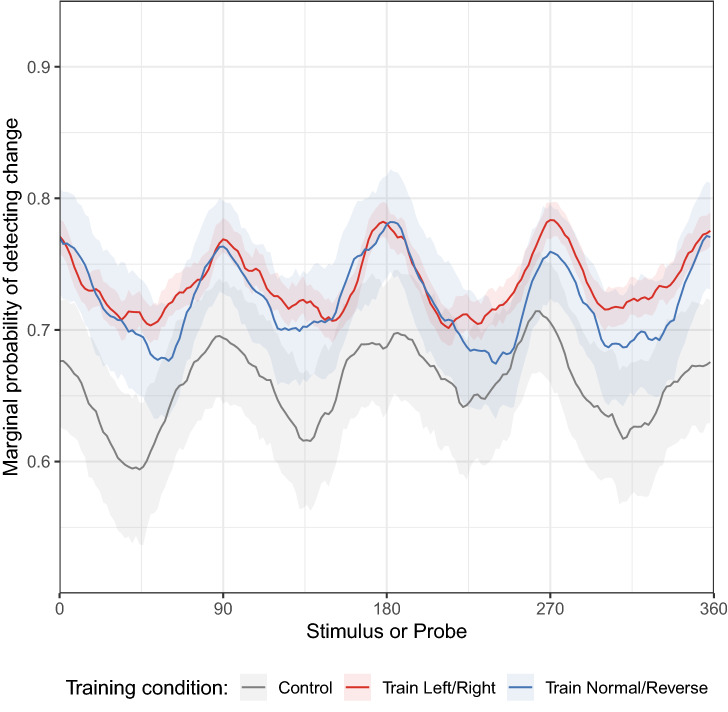
Mean probability of detecting a change, for a given memory stimulus or probe rake angle. Shaded regions around the lines indicate the 95% HDCI. (Figure generated using R version 4.0.3).

Lastly, our Bayesian model enables an analysis for change detection performance for all possible memory–probe combinations, even those not directly observed in the experimental data. An interesting analysis centers on the estimated probability for detecting a change for a given memory or probe, averaging over all possible paired stimuli. These results are illustrated in Fig. 6. The results show that human observers exhibited much greater sensitivity to visual changes when one of the two stimuli were close to horizontal or vertical rake angles. Figure 1b illustrates geologic faults corresponding to each of the peaks in perceptual discriminability shown in Fig. 6. This result is in keeping with the well-known oblique effect in visual perception, whereby visual discriminability is much greater for vertical and horizontal lines compared to oblique angles^10,30^. This further demonstrates that the study of VWM using complex stimuli can reveal rich structure in memory representations.

## Discussion

The experiment reported here provides direct empirical evidence that top-down categorical knowledge influences visual working memory (VWM) for complex visual information. Two types of results support this conclusion. First, it was found that participants trained to distinguish left/right lateral faults were more likely to detect a change in geologic scenes (depicted in memory and probe stimuli) when the scenes were mirror images along the left/right dimension. Similarly, participants trained to distinguish normal/reverse faults were more likely to detect a change when scenes were mirror images along the normal/reverse dimension. Second, participants were more likely to detect a change in stimuli that cross a fault boundary (e.g., the left/right lateral fault boundary) when they had been trained in that categorical distinction.

Taken in total, our results along with those reported by others^24–26^, suggest that the field of cognitive science may need to modify its conceptualization of VWM. VWM is typically regarded as a short-term memory store of objects’ visual properties, uninfluenced by objects’ semantic properties. If, however, VWM representations are influenced by semantic properties, then VWM should be reconceptualized so that it is closer to that of “conceptual short-term memory” (CSTM). In articles that have, to date, received relatively little attention, Potter^31,32^ argued for the existence of CSTM, and described it as a short-term memory store that is engaged unconsciously and rapidly, and represents current stimuli and their associated concepts from long-term memory, thereby providing a basis for the unreflective understanding characteristic of everyday experience. The use of CSTM, as opposed to the use of a VWM lacking top-down conceptual influence, would explain participants’ performance in our experiment.

Lastly, because participants were more likely to detect a change in stimuli that crossed a familiar (versus unfamiliar) category boundary, these results also highlight the task-dependent nature of VWM. People may be able to detect many visual properties, but memory (and perhaps other cognitive processes too) preferentially represent properties that seem at least potentially task relevant. For VWM and other capacity limited processes, preferential treatment of task-relevant information makes these processes more efficient.

## Methods

An archive of the complete dataset, along with supporting data analysis code, is available via the following URL: https://osf.io/gdz3t/.

### Experimental stimuli

As mentioned above, a geologic fault is a fracture or discontinuity in a volume of rock along which there has been displacement. Geoscience students commonly learn about both the spatial structures that characterize Earth’s crust as well as the causal processes that create these structures when studying structural geology^33^.

Consider the fault illustrated in Fig. 1a. It shows a fault plane separating two sections of Earth’s crust called fault blocks. In each figure, one of the blocks is labeled as the ‘hanging wall’ and the other is labeled as the ‘footwall’. There is a displacement in rock types on each side of the fault, indicating that the blocks have moved relative to each other. *Rake angle* is used to describe the direction of motion on a fault plane with respect to the strike (direction of the line formed by the intersection of a rock surface with a horizontal plane). In our analyses, values for rake angle range from 0$^\circ$ to 360$^\circ$ and are measured anticlockwise from the horizontal. (In the discipline of structural geology, the convention is to measure the rake angle from the strike ($0^\circ$) downwards to $90^\circ$. For angles exceeding $90^\circ$, the rake angle is measured from the opposite end of the strike downwards to $90^\circ$. Thus, in structural geology rake angles are always denoted as angles between 0$^\circ$ and $90^\circ$, with the appropriate strike direction indicated. Here, we use a different convention in order to more easily indicate the stimuli with a single number ranging between 0$^\circ$ and $360^\circ$).

To a trained geoscientist, faults possess a rich category structure, defined in part by their rake angle. Scientists measure rake angle as it provides important information regarding the causal forces responsible for motion along a fault. Figure 1b illustrates four categories of faults that differ in their rake angle: *Normal faults* (rake angles: 180$^\circ$–360$^\circ$) can be caused when two crustal blocks move away from each other as when the Earth’s crust is in a state of extension. *Reverse faults* (rake angles: 0$^\circ$–180$^\circ$) can form when the crust is in a state of compression, as is the case when two crustal blocks or tectonic plates collide. *Left lateral* (rake angles: 0$^\circ$–90$^\circ$ and 270$^\circ$–360$^\circ$) and *right lateral* (rake angles: 90$^\circ$–270$^\circ$) faults are caused by a lateral shift of fault blocks. These four basic fault categories are partially overlapping: a fault with a rake angle of $30^\circ$ is categorized as both a reverse fault and a left lateral fault.

Each visual stimulus in the experiment was a 4-s animation of a geologic fault. An animation showed a 360$^\circ$ perspective rotation around a fault in order to clearly depict its rake angle and physical structure. Stimuli differed solely in their rake angle (offset of the hanging wall relative to the footwall). Rake angle varied from 0$^\circ$ to 358$^\circ$ in 2$^\circ$ increments, resulting in 180 total stimuli used in the experiment. Consequently each stimulus was characterized by a single number, its rake angle.

### Participants

The experimental study was approved by the Research Review Board at Rensselaer Polytechnic Institute. Informed consent was obtained from all participants. Experiments were performed in accordance with relevant guidelines and regulations. In total, 537 participants participated in the experiment over the world wide web via the Amazon Mechanical Turk (MTurk) crowd-sourcing marketplace. Not all participants completed the task, and other participants were removed due to poor performance or apparent inattention to the task (details below). After removing these participants, 333 remained in the dataset (control condition: $N = 100$; train left/right: $N = 118$; train normal/reverse: $N = 115$).

Each participant received $3.25 for his or her participation. Interfacing with MTurk was facilitated through the psiTurk programming platform^34^ which was configured so that only individuals based in the United States were able to participate in the experiment.

### Procedure

Participants first completed a training session by watching a series of instructional videos pertaining to geologic faults. Participants were then randomly assigned to one of three different conditions. In the “train normal/reverse” condition, participants were trained to understand the distinction between normal and reverse geologic fault categories, but not the distinction between left and right lateral faults. In the “train left/right” condition, participants were trained in the distinction between lateral faults, but not in normal vs. reverse faults. In the control condition, participants received basic instruction on geologic faults (in common with the other conditions) but were not trained on any categorical distinctions between fault types.

Following training, participants completed a multiple-choice quiz testing their understanding of the instructions and geologic fault category knowledge (if trained). Participants were required to correctly answer the quiz questions before moving on to the main experiment.

The main experiment consisted of a visual change-detection task. On each trial, participants watched a sequence of two geologic fault animations: a memory stimulus, followed by a blank retention interval (lasting 1.5 s), followed by a probe stimulus. The memory stimulus was randomly sampled from all possible rake angles. Half of the trials were ‘same trials’, meaning that the probe in a trial was identical to the memory stimulus, except shown using a different animation (a different starting perspective and different animation rotation direction). The remaining half of trials were ‘change trials’ where the probe was uniformly sampled from the stimulus space excluding the memory stimulus. The task for participants was to determine whether the memory stimulus and probe were the same or different in terms of their fault structure. Responses were indicated by pressing one of two keys on the keyboard.

Each participant completed a total of 50 change detection trials. It was discovered post hoc that some participants with slow internet connections experienced lag in viewing the fault animations. Consequently, the following exclusion criteria were adopted: we eliminated individual trials with lag greater than 2.5 s, eliminated trials with response time (RT) greater than 5 s, and eliminated entire participants with fewer than 25 remaining trials meeting both lag and RT criteria. In addition, we eliminated subjects who did not reach at least 60% correct across all 50 trials. Applying these exclusion criteria resulted in a total of 16,004 trials from 537 participants.

### Data analysis: Gaussian Markov Random Field (GMRF)

In a typical visual change detection experiment, each memory–probe combination is presented a number of times, and performance is characterized in terms of the probability that an observer detects a change of a given magnitude. A challenge with this approach arises in the current experiment due to the large number of possible memory–probe combinations (180 potential rake angles for memory stimulus $\times$ 180 potential rake angles for probe stimulus = 32,400 possible combinations). Of all possible combinations, roughly 10% were sampled and shown to participants in our experiment, and only about 1% were sampled more than once. Thus, due to the sparsely sampled stimulus space, computing the probability of detecting a change for a given memory–probe combination is not straightforward.

To address this limitation we developed a Bayesian analysis technique based on Gaussian Markov random fields (GMRFs). A GMRF is an undirected probabilistic graphical model, in which observations are defined over a graph (or lattice), that captures spatial dependencies between neighboring nodes in the graph. GMRFs have been extensively used in modeling spatial statistics such as those that arise in disease mapping, geology, and image analysis^28,35^. For our purposes, GMRFs are a convenient (and mathematically principled) way of implementing the assumption (in Bayesian parlance, a “prior belief”) that participants should have similar probabilities of detecting changes in similar memory–probe combinations. GMRFs are commonly used to study large, real-world problems because when a GMRF assumes that neighboring nodes have similar values, it can share statistical strength between spatially neighboring observations such as infectious disease rates in neighboring counties or, in the case studied here, change detection performance between similar memory–probe combinations.

**Figure 7:**
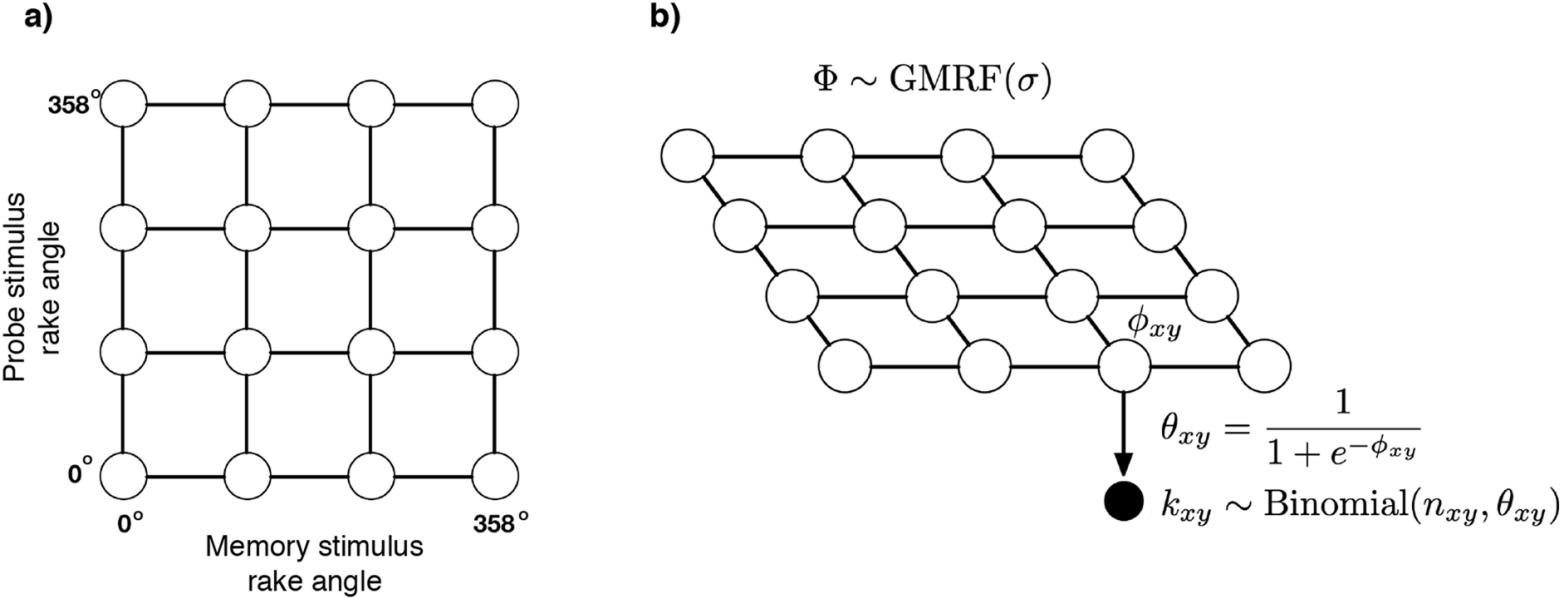
Gaussian Markov Random Field (GMRF) used to analyze visual change detection performance. (**a**) A 2D lattice defined over all memory–probe pairs. The figure shows a $4 \times 4$ lattice for simplicity, the actual analysis used a $180 \times 180$ lattice. (**b**) A probabilistic graphical model connecting latent Gaussian values defined by a GMRF to the experimentally-measured change detection data. (Figure generated using Adobe Illustrator 2021).

Specifying a GMRF requires specifying a conditional probability distribution of a node given its neighbors in a graph structure. In our case, we defined a $180 \times 180$ two-dimensional lattice corresponding to the memory–probe structure of our experiment, with each node in the lattice corresponding to an individual memory–probe combination (Fig. 7a). Each node in the lattice has an associated real value that has a Gaussian prior probability distribution. For a single node defined by memory stimulus *x* and probe *y*, the value associated with that node is denoted $\phi _{xy}$, as shown in Fig. 7b.

A key feature of our GMRF is that nodes that are directly connected in the network are assumed to have similar values. This is implemented by defining a prior over the *difference* between node values. In other words, for two nodes $\phi _a$ and $\phi _b$ connected by an edge in the lattice,

1 $$\begin{aligned} \left( \phi _a - \phi _b \right) \sim \text {Normal}(0, \sigma ). \end{aligned}$$

The parameter $\sigma$ determines the amount of spatial correlation between neighboring nodes. In our case this parameter was fixed to the value 2. Defining a prior over differences between neighboring nodes in turn defines a prior over the vector of Gaussian values for the entire graph:

2 $$\begin{aligned} p(\Phi ) \propto \prod _{i\sim j} \mathrm {exp}\left( -\frac{1}{2 \sigma ^2} \left( \phi _i - \phi _j \right) ^2 \right) , \end{aligned}$$

where the product is taken over all pairs of nodes *i* and *j* that are connected by an edge in the graph. Random samples from this prior tend to be relatively “smooth” (i.e., nearby locations have similar probabilities). For futher mathematical background on GMRFs, see^28^.

To use our GMRF in Bayesian inference, we must also specify a likelihood function, or a means of connecting the latent Gaussian values with experimentally-measured data. To that end, we defined the probability of a human participant reporting ‘change’ given a memory stimulus *x* and probe *y* as $\theta _{xy}$, and modeled this as a logistic function of the corresponding Gaussian value in the graph:

3 $$\begin{aligned} \theta _{xy} = \frac{1}{1 + e^{-\phi _{xy}}}. \end{aligned}$$

If a particular memory–probe combination was presented $n_{xy}$ times, and was reported to be a ‘change’ trial $k_{xy} \le n_{xy}$ times, then according to our analysis, $k_{xy}$ has a Binomial distribution:

4 $$\begin{aligned} k_{xy} \sim \text {Binomial}(n_{xy}, \theta _{xy}). \end{aligned}$$

As applied to our experiment, edges were defined between all memory–probe combinations differing by one increment in rake angle (2$^\circ$). Because the stimulus space is circular, the edges of the lattice also ‘wrapped around’ (not shown in Fig. 7 for simplicity).

The GMRF prior distribution and the likelihood function (Eqs. 3 and 4) define the Bayesian model used to analyze our experimental data. Bayesian inference was performed by implementing this model in the Stan probabilistic programming language^36^. Stan utilizes Markov Chain Monte Carlo (MCMC) techniques to approximate a posterior distribution via stochastic sampling. The resulting posterior indicates the estimated probability that human observers will detect a change for a given memory–probe combination, even when the stimulus space is only sparsely sampled. For our analyses, we used five MCMC chains, performing 2000 warmup or burn-in iterations, and collecting 500 posterior samples from each chain.
